# A New Indole Alkaloid from the Toad Venom of *Bufo bufo gargarizans*

**DOI:** 10.3390/molecules21030349

**Published:** 2016-03-16

**Authors:** Ying-Hui Dai, Bo Shen, Ming-Yu Xia, An-Dong Wang, Yu-Lin Chen, Dong-Chun Liu, Dong Wang

**Affiliations:** 1 School of Traditional Chinese Materia Medica, Shenyang Pharmaceutical University, Shenyang 110016, Liaoning, China; yhdai2008@aliyun.com (Y.-H.D.); sb.prove@163.com (B.S.); wangandong19891220@163.com (A.-D.W.); cyl855@163.com (Y.-L.C.); liudongchun@outlook.com (D.-C.L.); 2 School of Life Science and Biopharmaceutics, Shenyang Pharmaceutical University, Shenyang 110016, Liaoning, China; xmywd@vip.sina.com; 3 Laboratory of China-Korea Molecular Pharmacognosy, Shenyang Pharmaceutical University, Shenyang 110016, Liaoning, China

**Keywords:** *Bufo bufo gargarizans*, toad venom, bufobutarginine, cytotoxic activity

## Abstract

A new indole alkaloid named bufobutarginine (**1**), along with three known bufotenines, namely, serotonin (**2**), bufotenidine (**3**), and bufotenine (**4**), were isolated from the water extract of toad venom. Their structures were elucidated by spectral methods. This is the first time that arginine has been found to be involved in the biosynthesis of bufotenines in parotid of toad. The cytotoxic activities of these compounds have been assayed against A375 and A549 cell lines by the MTT method; however, they showed no cytotoxic activities.

## 1. Introduction

Toad venom is the dry secretions derived from giant toads, including *Bufo bufo gargarizans* Cantor and *B. melanostrictus* Schneider. In traditional Chinese medicine, toad venom possesses a wide range of biological effects and has long been used for the treatment of heart failure, tumors, sores, and pains in clinical settings [1,2]. Toad skin is another crude drug from *B. bufo gargarizans* with similar chemical components and the same biological effect as toad venom [2,3]. Their major active constituents are bufogenins, bufotoxins, and bufotenines. Among them, the bufogenins, a kind of liposoluble constituents, have been known to be a primary active substance, which is attributed to their significant biological activities such as cardiotonic, hypertensive, and antitumor effects [3,4]. However, the preparations of toad venom or toad skin used as antitumor agents in clinics are usually their water-soluble components such as the Chan’su injection and the Cinobufacini injection, each of which contain only trace amounts of bufogenins [5]. Based on the facts mentioned above, we presumed that the water-soluble components of toad venom might possess a strong antiproliferative activity. In order to further investigate the antitumor material basis of toad venom, we studied the water-soluble components of toad venom. In this paper, we describe the isolation and structural elucidation of a new indole alkaloid, along with three known compounds. Their structures were established by extensive spectroscopic data analysis and comparison with literature values. Furthermore, the cytotoxic activities of all the isolated compounds were evaluated.

## 2. Results and Discussion

### 2.1. Structure Elucidation

**Figure 1:**
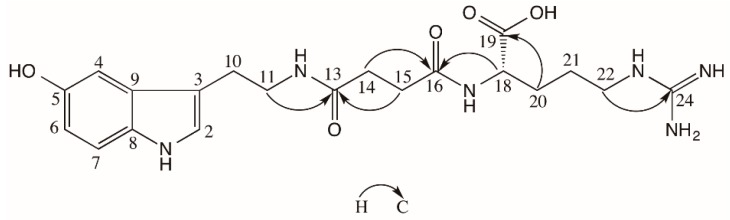
Key HMBC correlations of compound **1**.

Compound **1** was obtained in the form of pale yellow crystals. The molecular formula C_20_H_28_N_6_O_5_ was established by HR-ESI-MS spectrometry at *m*/*z* 433.2193 [M + H]^+^ (calculated 433.2199). Hydrolysis with 6 M hydrochloric acid provided arginine, which was identified by TLC with l-arginine standard [6,7]. In the ^1^H-NMR (600 MHz, D_2_O) spectrum of **1**, signals at δ_H_ 7.11 (1H, s, H-2), 7.00 (1H, d, *J* = 2.1 Hz, H-4), 6.75 (1H, dd, *J* = 8.7, 2.1 Hz, H-6), and 7.30 (1H, d, *J* = 8.7 Hz, H-7) indicated a typical 3,5-disubstituted indole moiety. Combined with two methylene signals at 3.36 (2H, t, *J* = 6.8 Hz, H-11) and 2.81 (2H, t, *J* = 6.8 Hz, H-10), it was suggested that **1** is a derivative of serotonin. The ^13^C-NMR (150 MHz, CD_3_OD) spectrum showed twenty carbon signals. Ten of them were confirmed by comparing them with the NMR data of serotonin as δ_C_ 24.8 (C-10), 40.1 (C-11), 102.3 (C-4), 111.1 (C-3), 111.2 (C-6), 111.6 (C-7), 123.3 (C-2), 128.0 (C-9), 131.6 (C-8), and 149.4 (C-5) [8]. In addition, there were six carbon signals, δ_C_ 24.7 (C-21), 29.5 (C-20), 40.7 (C-22), 54.3 (C-18), 157.0 (C-24), 177.6 (C-19), which were almost the same in comparison with the ^13^C-NMR data of arginine [9]. The signal at δ_H_ 4.07 (1H, dd, *J* = 4.8, 8.3 Hz, H-18) in the ^1^H-NMR spectrum also supported the existence of arginine moiety in **1**. In the high field of the ^1^H-NMR spectrum two methylene proton signals were observed at δ_H_ 2.39 (4H, m), indicating that the two methylenes were in a similar chemical surroundings influenced by the deshielding effect. Meanwhile, the carbon signals of the succinyl moiety were observed in the ^13^C-NMR spectrum at δ_C_ 31.1, 31.2 (C-14, 15), 172.9 (C-16), and 173.5 (C-13), so it is confirmed that the succinyl moiety was also one piece of the structure of **1**. The HMBC correlations between H-11 (δ_H_ 3.36) and C-13 (δ_C_ 173.5), and between H-18 (δ_H_ 4.07) and C-16 (δ_C_ 172.9), indicated that the succinyl moiety was a bridge connecting the serotonin and arginine moiety by N-12 and N-17, respectively (Figure 1). The NMR data of **1** is shown in Table 1. The hydrolysate of **1** by 6 M hydrochloric acid was analyzed on a chiral HPLC column to determine the absolute stereochemistry of arginine moiety. Only l-arginine was detected in the hydrolysate of **1**. Thus, the structure of **1** was established as 4-((2-(5-hydroxy-1*H*-indol-3-yl)ethyl)animo)-4-oxobutanoyl)-l-arginine, named bufobutarginine. Up to now, the arginine moiety has only been found in bufogenins to form bufotoxins [2]. Therefore, it is the first time that arginine has been found to be involved in the biosynthesis of bufotenines.

The known compounds were readily identified as serotonin (**2**), bufotenidine (**3**), and bufotenine (**4**) by comparing NMR spectral data with those reported in the literature [8].

### 2.2. Cytotoxic Activities

The *in vitro* cytotoxicities against two human carcinoma cell lines (A549 and A375) of **1**–**4** were examined. However, none of them exhibited cytotoxic effects, even with the concentration of 200 μM. The max inhibitions against A549 and A375 were 2.54% and 25.58% , respectively. Up to now, only three bufotenines—bufobutanoic acid, bufopyramide, and bufothionine—showed cytotoxic activities against the murine leukemia cell line P388, human hepatocellular carcinoma cell lines SMMC-7721, and BEL-7402 [10,11]. Compound **1** is an arginine derivative of bufobutanoic acid, but **1** did not exhibit cytotoxic activities against carcinoma cells. The free carboxy moiety is possibly the key group for cytotoxic activity of bufobutanoic acid, and the arginine moiety might play an important role in decreasing the toxicity of secretions. Compound **1** might be a prodrug of bufobutanoic acid with potential cytotoxic activity.

## 3. Experimental Section

### 3.1. General Information

Melting points were measured with MEL-TEMP micromelting point apparatus (Shanghai Yidian Analytical Instrument Company, Shanghai, China). Optical rotation was measured on a JASCOP-1020 Polarimeter (Jasco Co., Tokyo, Japan). UV spectra were recorded on a Shimadzu UV-1700 Spectrophotometer (Shimadzu Co., Kyoto, Japan). IR spectra were recorded on a Bruker IFS 55 FTIR spectrometer on KBr pellets (Bruker Co., Karlsruhe, Germany). CD spectrum was recorded by a MOS 450 detector (Bio-Logic Co., Claix, France). HR-ESI-MS data were recorded on a Waters Xevo G2 Q-TOF mass spectrometer (Waters Co., Milford, MA, USA). NMR spectra were taken with Bruker ARX-600 spectrometer (chemical shift values are presented as δ values with TMS as the internal standard; Bruker Co., Billerica, MA, USA). Column chromatography was performed on ODS (50 μm, YMC, Komatsu, Japan), and Sephadex LH-20 (40–70 μm, Amersham Pharmacia Biotech AB, Uppsala, Sweden). TLC was conducted on silica gel GF_254_ (Marine Chemical Factory, Qingdao, China) plates.

### 3.2. Materials

The toad venom was collected in Linyi, Shandong Province, China, in March 2010. Each of the crude materials was identified by the Associate Professor Dong Wang from Shenyang Pharmaceutical University as toad venom of *B. bufo gargarizans*. Human malignant melanoma cells A375 and human lung adenocarcinoma epithelial cells A549 were purchased from the American Type Culture Collection (ATCC, Rockville, MD, USA).

### 3.3. Extraction, Isolation and Characterization

The dried toad venom (150 g) was ground into a coarse powder and extracted with dichloromethane (7 × 1.5 L) under reflux, and the extract was concentrated *in vacuo* to obtain the dichloromethane extract (21 g). The residue (100 g) was extracted 5 times with 1 L of water via an ultrasonator (200 W, 59 kHz, 30 min). The water was evaporated under vacuum to obtain 90 g of crude water extract. The crude water extract was suspended in 1 L of water and partitioned with 1 L of *n*-BuOH. EtOH was added into the water phase to a final concentration of 75% (*v*/*v*), and then kept for 12 h at 4 °C. The filtrate was concentrated under vacuum to give the dry residue (40 g). A part of the residue (18 g) was subjected to ODS column eluting with MeOH–H_2_O from *v*/*v* 32:68 to 40:60 to yield 2 fractions. Fraction 1 was further separated by PTLC (20 × 20 cm, 0.5 mm), developed with *n*-BuOH–HOAc–H_2_O (4:1:5), and purified by a Sephadex LH-20 column eluted with 30% (*v*/*v*) MeOH to give compound **2** (6.8 mg) and compound **3** (6.6 mg). Fraction 2 was subjected to PTLC, developed with *n*-BuOH–HOAc–H_2_O (4:1:5), and then to a Sephadex LH-20 column eluted with 40% (*v*/*v*) MeOH to obtain compound **1** (4.5 mg) and compound **4** (4.7 mg).

*Bufobutarginine* (**1**): Yellow crystals (MeOH); m.p. 194–196 °C; HR-ESI-MS: *m*/*z* 433.2193 [M + H]^+^ (calcd. for C_20_H_29_N_6_O_5_, 433.2199); ${\left[\alpha \right]}_{D}^{20}$ − 62.2 (*c* 0.9, H_2_O); UV (H_2_O) λ_max_ (log ε): 217 (4.38), 281 (3.56), 380 (3.52) nm; IR (KBr) ν_max_: 3385, 2930, 1633, 1578, 1468, 1456, 1384, 1188 cm^−1^; ^1^H NMR and ^13^C NMR spectral data, see Table 1; CD (H_2_O): Δε_194nm_ − 4.14, Δε_202nm_ + 1.82, Δε_212nm_ − 3.43.

### 3.4. Acid Hydrolysis of Compound **1**

Compound **1** (1 mg) was heated with 6 M HCl (0.5 mL) in a sealed tube at 100 °C for 8 h. A portion of the resulting solution was subjected to TLC on silica gel GF_254_ with *n*-butanol–HOAc–EtOH–H_2_O (4:1:1:2) as a developing solvent. Ninhydrin reagent was used for the detection of amino acids on the TLC plate [6,7].

### 3.5. HPLC Analysis

The hydrolysate of **1** was analyzed using Waters 2695 HPLC with a 2478 UV detector. HPLC experiments were performed on Chirex 3126 column (250 × 4.0 mm, 5 μm, Phenomnex, Torrance, CA, USA) at a flow rate of 1.0 mL/min eluted with 1 mM CuSO_4_. Column temperature was maintained at room temperature. Detection was carried out at 254 nm. l- and d-arginine were products of Aladdin (China).

### 3.6. Cytotoxic Assays 

The *in vitro* cytotoxic activities test on two human tumor cell lines were performed using MTT methods in a 96-multiwell microtiter integrated system. Compounds **1**–**4** were dissolved in DMSO to make stock solutions, then diluted in cell culture medium at different concentrations, and used immediately. The positive control was 5-fluorouracil (5-FU) dissolved in the same solution. In all assays, the final concentrations of DMSO in the culture medium were less than 0.01%. The cells were cultured in a RPMI-1640 medium (Gibco, Grand Island, NY, USA) and supplemented with 10% fetal bovine serum (FBS) (Dalian Biological Reagent Factory, Dalian, China) and 0.03% l-glutamine (Gibco) at 37 °C in 5% CO_2_. Tumor cells were seeded at 5 × 10^4^ cells/well in 96-well plates (Nunc, Roskilde, Denmark). After overnight incubation, different concentrations of **1**–**4** were added, and the final concentrations were 0.1, 1, 10, 100, and 200 μM, respectively. Incubated for 0, 12, 24, and 48 h, cell growth was measured at different time points in the 3-(4,5-dimethylthiazol-2-yl)-2,5-diphenyltetrazolium bromide (MTT) assay with a plate reader (Tecan, Grödig, Austria) [12]. The inhibition ratio (%) was calculated using the following formula:

(1) $$\mathrm{Inhibiton ratio}\;(\%)=\left(1-\frac{\mathrm{mean survival of treated group}}{\mathrm{mean survival of control}}\right)\times 100\%$$

## Figures and Tables

**Table 1:** The ^1^H-NMR (600 MHz, D_2_O), ^13^C-NMR (150 MHz, CD_3_OD) spectroscopic data for bufobutarginine (**1**) with HMBC correlations.

Position	δ_C_, Type	δ_H_ (*J* in Hz)	HMBC ^a^
2	123.3, CH	7.11, s	3, 8, 9, 10, 11
3	111.1, C		
4	102.3, CH	7.00, d (2.1)	3, 5, 8
5	149.4, C		
6	111.2, CH	6.75, dd (2.1, 8.7)	4, 5, 8
7	111.6, CH	7.30, d (8.7)	4, 5, 9
8	131.6, C		
9	128.0, C		
10	24.8, CH_2_	2.81, t (6.8)	2, 3, 11, 13
11	40.1, CH_2_	3.36, t (6.8)	3, 10, 13
13	173.5, C		
14	31.2, CH_2_	2.39, m	16
15	31.1, CH_2_	2.39, m	13
16	172.9, C		
18	54.3, CH	4.07, dd (4.8, 8.3)	16, 19, 20, 21
19	177.6, C		
20	29.5, CH_2_	1.56, m	18, 19, 21, 22
		1.71, m	
21	24.7, CH_2_	1.43, m	18, 20, 22
22	40.7, CH_2_	2.99, t (6.9)	20, 21, 24
24	157.0, C		

^a^ HMBC correlations are from proton(s) stated to the indicated carbon.
